# Anti-Inflammatory Potential of Ethyl Acetate Fraction of *Moringa oleifera* in Downregulating the NF-κB Signaling Pathway in Lipopolysaccharide-Stimulated Macrophages

**DOI:** 10.3390/molecules21111452

**Published:** 2016-10-31

**Authors:** Palanisamy Arulselvan, Woan Sean Tan, Sivapragasam Gothai, Katyakyini Muniandy, Sharida Fakurazi, Norhaizan Mohd Esa, Abdullah A. Alarfaj, S. Suresh Kumar

**Affiliations:** 1 Laboratory of Vaccines and Immunotherapeutics, Institute of Bioscience, Universiti Putra Malaysia, Serdang 43400, Selangor, Malaysia; wansen.0801@gmail.com (W.S.T.); gothai_86@yahoo.com (S.G.); katyakyini_2708@hotmail.com (K.M.); sharida.fakurazi@gmail.com (S.F.); 2 Department of Human Anatomy, Faculty of Medicine and Health Sciences, Universiti Putra Malaysia, Serdang 43400, Selangor, Malaysia; 3 Department of Nutrition and Dietetics, Faculty of Medicine and Health Sciences, Universiti Putra Malaysia, Serdang 43400, Selangor, Malaysia; nhaizan@upm.edu.my; 4 Department of Botany and Microbiology, King Saud University, Riyadh 11451, Saudi Arabia; aalarfajj@ksu.edu.sa; 5 Department of Medical Microbiology and Parasitology, Faculty of Medicine and Health Sciences, Universiti Putra Malaysia, Serdang 43400, Selangor, Malaysia; sureshkudsc@gmail.com

**Keywords:** inflammation, proinflammatory cytokines, RAW264.7 cells, inflammatory mediators, IκBα

## Abstract

In the present investigation, we prepared four different solvent fractions (chloroform, hexane, butanol, and ethyl acetate) of *Moringa oleifera* extract to evaluate its anti-inflammatory potential and cellular mechanism of action in lipopolysaccharide (LPS)-induced RAW264.7 cells. Cell cytotoxicity assay suggested that the solvent fractions were not cytotoxic to macrophages at concentrations up to 200 µg/mL. The ethyl acetate fraction suppressed LPS-induced production of nitric oxide and proinflammatory cytokines in macrophages in a concentration-dependent manner and was more effective than the other fractions. Immunoblot observations revealed that the ethyl acetate fraction effectively inhibited the expression of inflammatory mediators including cyclooxygenase-2, inducible nitric oxide synthase, and nuclear factor (NF)-κB p65 through suppression of the NF-κB signaling pathway. Furthermore, it upregulated the expression of the inhibitor of κB (IκBα) and blocked the nuclear translocation of NF-κB. These findings indicated that the ethyl acetate fraction of *M. oleifera* exhibited potent anti-inflammatory activity in LPS-stimulated macrophages via suppression of the NF-κB signaling pathway.

## 1. Introduction

Inflammation is a natural immunological response to injuries and infections as a result of harmful stimuli, including microbial pathogens, tissue injury, and immunological cross interactions in the body [1]. Severe and chronic inflammation may lead to the development of chronic inflammation-associated diseases and disorders such as diabetes, cancer, autoimmune diseases, cardiovascular diseases, sepsis, colitis, and arthritis [2,3]. Numerous inflammatory mediators including proinflammatory cytokines, chemokines, free radicals, and certain enzymes are involved in this inflammatory process mediated by activated immune cells such as monocytes and macrophages [4]. Macrophages play important roles in the initiation and modulation of host defense mechanisms through the production of proinflammatory mediators (e.g., nitric oxide, cytokines, and prostaglandin E2 (PGE2) [5]. Various signal transduction pathways are involved in the inflammatory process and contribute to both acute and chronic inflammation. Nuclear factor (NF)-κB is central to inflammatory responses and induces the expression of key inflammatory genes. In addition, it facilitates the pathogenesis of various chronic inflammatory diseases and disorders [6]. Therefore, downregulation of the NF-κB signaling pathway is one of the major targets to alleviate chronic inflammation and its associated diseases.

Effective anti-inflammatory drugs such as steroids and nonsteroidal anti-inflammatory therapeutics are currently available for the treatment of acute and chronic inflammatory diseases. Nevertheless, long-term use of these anti-inflammatory drugs can cause various adverse effects to human health. Thus, it remains a challenge for biomedical researchers and pharmaceutical players to develop drugs with greater efficacy and minimal toxicity to treat chronic inflammation and its associated diseases [7].

Natural products play a key role in the drug development process, where they are used as sources from which many pharmaceutical drugs are derived. *Moringa oleifera* (family Moringaceae) is a highly nutritious medicinal plant that is widely distributed in tropical and subtropical countries, like those in the Indian subcontinent [8]. It has been used as a folk medicine against numerous diseases and disorders. Among all the edible parts of *M. oleifera*, the leaves and its active extracts are excellent sources of natural antioxidants owing to the presence of ascorbic acid, β-carotene, flavonoids, phenolics, and carotenoids [9]. Recently, we reported that *M. oleifera* leaf extract-supplemented animal feed enhanced the antioxidant status of chickens [10]. Previously, we have investigated the various medicinal properties of *M. oleifera* leaf extract including its hepatoprotective, wound healing, and antioxidant activities, and have identified several pharmacologically active compounds from its extracts [11,12,13,14]. Furthermore, other researchers have demonstrated its antidiabetic, anticancer, antiulcer, antibacterial, and immunomodulatory properties [15,16,17,18,19]. Nevertheless, its cellular and molecular mechanisms of action against various inflammation-associated diseases are still unclear. 

In the present study, we investigated the inhibitory effects of several *M. oleifera* fractions and identified the possible mechanisms of action in lipopolysaccharide (LPS)-stimulated macrophages. Our findings clearly indicated that the ethyl acetate fraction of *M. oleifera* significantly inhibited NF-κB signaling pathways through downregulation of proinflammatory mediators and upregulation of inhibitor of κB (IκB)α expression.

## 2. Results

### 2.1. Cytotoxicity of M. oleifera Fractions in RAW264.7 Cells

**Figure 1:**
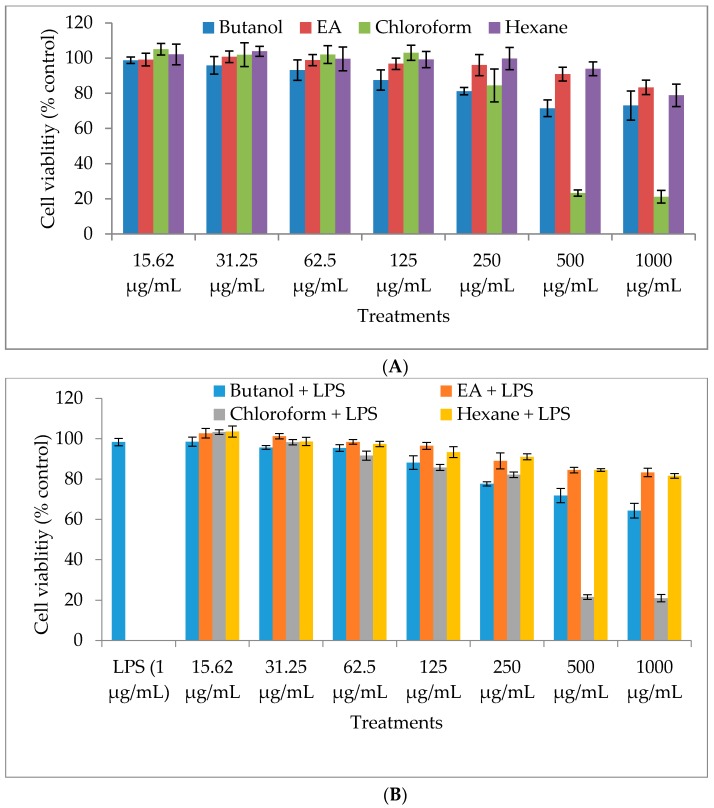
Effect of different solvent fractions (butanol (BUT), ethyl acetate (EA), chloroform (CHO), and hexane (HEX)) of *M. oleifera* extract on cell viability of RAW264.7 macrophages. The macrophages were treated with various concentrations of the fractions without lipopolysaccharide (LPS) (**A**) and with LPS (**B**) incubated for 24 h. After the incubation period, cell viability was determined by MTT (3-(4,5-dimethylthiazol-2-yl)-2,5-diphenyltetrazolium bromide) assay. Values are expressed as the percentage viability of the control cells. All experiments were conducted in triplicate and results are expressed as mean ± SD.

To investigate the cytotoxic potential of *M. oleifera* fractions (butanol, ethyl acetate, chloroform, and hexane) in RAW264.7 cells, the MTT (3-(4,5-dimethylthiazol-2-yl)-2,5-diphenyltetrazolium bromide) assay was performed to measure cell viability after 24 h treatment with the fractions. The viability of the treated cells decreased in a dose-dependent manner (Figure 1). In this assay, we used seven different concentrations of each fraction (15.62, 31.25, 62.5, 125, 250, 500, and 1000 µg/mL) and found that they showed significant cytotoxicity at high concentrations, particularly the chloroform fraction (80% cytotoxicity at 500 and 1000 µg/mL). All fractions were nontoxic to the cells at lower concentrations; therefore, the concentrations of the solvent fractions for further anti-inflammatory mechanistic experiments were set at 50, 100, and 200 µg/mL.

### 2.2. Effect of M. oleifera Fractions on Nitric Oxide (NO) Production in LPS-Stimulated RAW264.7 Cells

**Figure 2:**
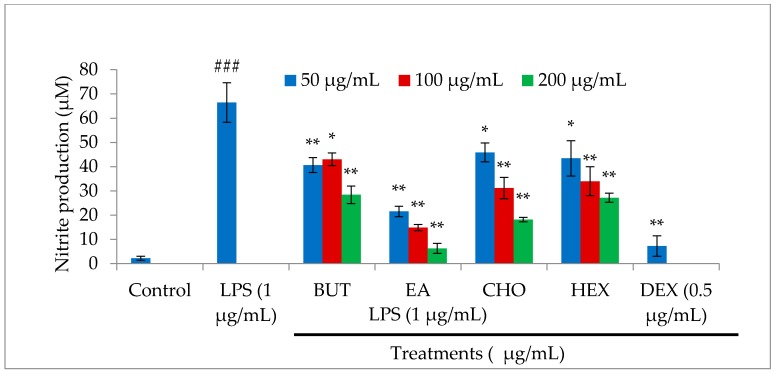
Effect of different solvent fractions (butanol, ethyl acetate, chloroform, and hexane) of *M. oleifera* extract on nitric oxide (NO) production in LPS-stimulated macrophages. Macrophages were treated with LPS in the absence or presence of the fractions at various concentrations (50, 100, and 200 µg/mL) for 24 h. After the incubation period, cell culture supernatants were harvested and NO production was determined by Griess reagent. Values are expressed as described in the Methods section. All experiments were conducted in triplicate and results are expressed as mean ± SD. ^###^ *p* < 0.001 vs. control; ** *p* < 0.01 and * *p* < 0.05 vs. LPS treatment.

Nitric oxide is one of the key inflammatory mediators linked with various acute and chronic inflammation-associated diseases. In macrophages, NO production is significantly increased upon LPS-stimulation. The level of NO production was estimated in cell culture supernatant after the treatment of LPS-stimulated macrophages with *M. oleifera* fractions using Griess reagent. As shown in Figure 2, LPS treatment significantly stimulated NO production, while treatments with *M. oleifera* fractions and dexamethasone (positive control drug) significantly inhibited NO production in a dose-dependent manner. The ethyl acetate fraction showed a stronger inhibitory effect on NO production than the other fractions, indicating that it has the most significant anti-inflammatory potential among all fractions.

### 2.3. Effect of M. oleifera Fractions on Proinflammatory Cytokine and PGE2 Production in LPS-Stimulated RAW264.7 Cells

**Figure 3:**
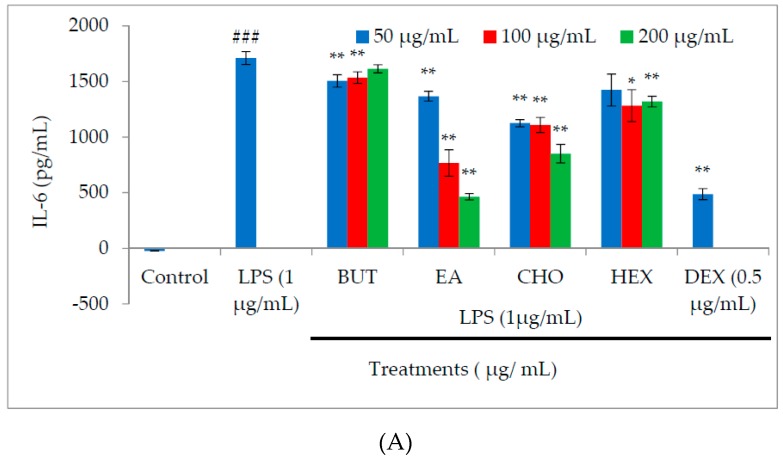

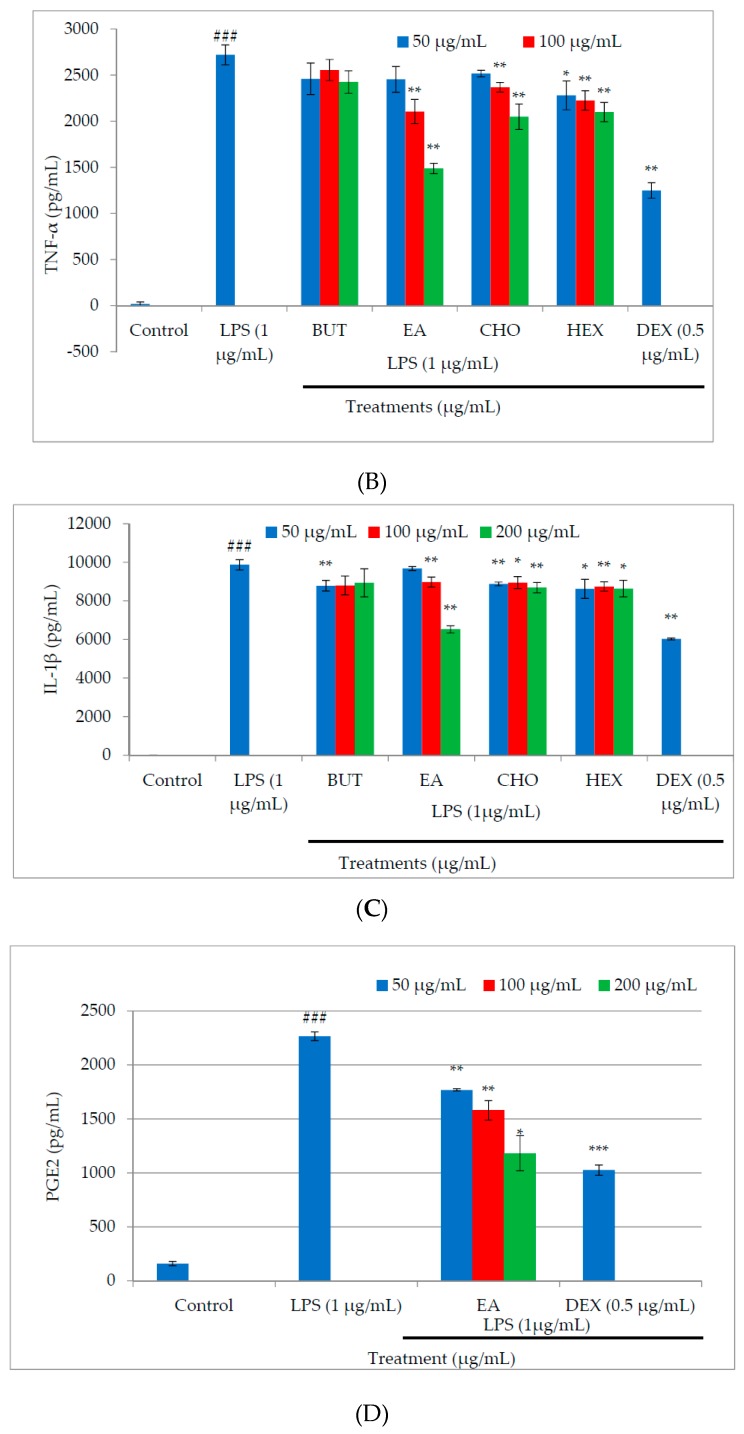
Effect of different solvent fractions (butanol, ethyl acetate, chloroform, and hexane) of *M. oleifera* extract on interleukin (IL)-6 (**A**), tumor necrosis factor (TNF)-α (**B**), IL-1β (**C**), and prostaglandin E2 (PGE2) (**D**) production in LPS-stimulated macrophages. Macrophages were treated with LPS in the absence or presence of the fractions at various concentrations (50, 100, and 200 µg/mL) for 24 h. After the incubation period, cell culture supernatants were harvested, and IL-6, TNF-α, IL-1β, and PGE2 production was determined by ELISA. Values are expressed as described in the methods section. All experiments were conducted in triplicate and results are expressed as mean ± SD. ^###^ *p* < 0.001 vs. control; *** *p* < 0.001, ** *p* < 0.01, and * *p* < 0.05 vs. LPS treatment.

Macrophages are a key source of proinflammatory cytokines, and it is well established that exposure to LPS induces the production of both proinflammatory and anti-inflammatory cytokines that lead to inflammation and its associated diseases. The anti-inflammatory effects of *M. oleifera* fractions on the production of proinflammatory cytokines and PGE2 were determined in cell culture supernatant using enzyme-linked immunosorbent assay (ELISA). Treatment of LPS-stimulated macrophages with the fractions led to concentration-dependent inhibition of interleukin (IL)-6, tumor necrosis factor (TNF)-α, and IL-1β production (Figure 3A–C). The inhibitory effect of the ethyl acetate fraction was more potent than that of the other fractions. Among the cytokines, the production of IL-6 was the most strongly suppressed by the ethyl acetate fraction. As shown in Figure 3D, treatment with the ethyl acetate fraction also significantly reduced the production of PGE2 in a dose-dependent manner.

### 2.4. Effect of M. oleifera Ethyl Acetate Fraction on Inducible Nitric Oxide Synthase (iNOS) and Cyclooxygenase (COX)-2 Productions in LPS-Stimulated RAW264.7 Cells

**Figure 4:**
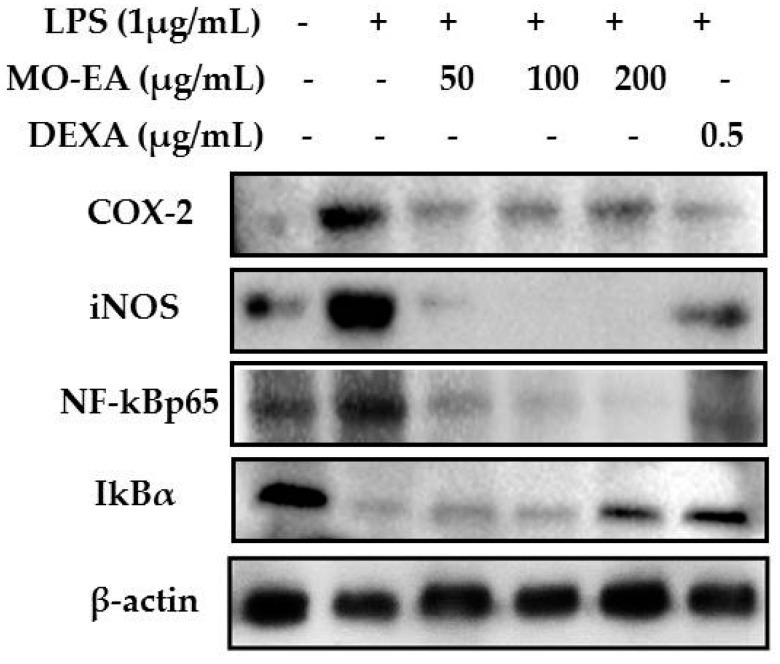
Effect of the ethyl acetate fraction of *M. oleifera* extract on the expression of inflammatory mediators, cyclooxygenase (COX)-2, inducible nitric oxide synthase (iNOS), nuclear factor (NF)-κB p65, and inhibitor of κB (IκBα) in LPS-stimulated macrophages. Macrophages were treated with LPS in the absence or presence of the ethyl acetate fraction at various concentrations (50, 100, and 200 µg/mL) for 24 h. After the incubation period, proteins were extracted from treated cells and subjected to electrophoresis, and inflammatory mediator expression was detected by Western blots. All experiments were conducted in triplicate and images shown are representatives of triplicate experiments.

Expression of inflammatory mediators is primarily induced by LPS in macrophages and other immune cells. We investigated the effects of the ethyl acetate fraction on the inflammatory mediators iNOS and COX-2, which are responsible for the production of NO and PGE2, using immunoblot analysis. Figure 4 shows that the protein expression of iNOS and COX-2 was upregulated in LPS-stimulated macrophages, but not in the untreated cells. The ethyl acetate fraction effectively downregulated the expression of iNOS and COX-2 in a dose-dependent manner.

### 2.5. Effect of M. oleifera Ethyl Acetate Fraction on IκBα Degradation and NF-κB Translocation in LPS-Stimulated RAW264.7 Cells

The transcription factor NF-κB plays an important role during the inflammation process in immune cells, particularly in macrophages. We investigated the effect of the ethyl acetate fraction on LPS-stimulated degradation of IκBα in macrophages (Figure 4). The expression of IκBα was considerably downregulated after LPS exposure. However, treatment with ethyl acetate fraction and dexamethasone markedly inhibited the degradation of IκBα in a concentration-dependent manner.

**Figure 5:**
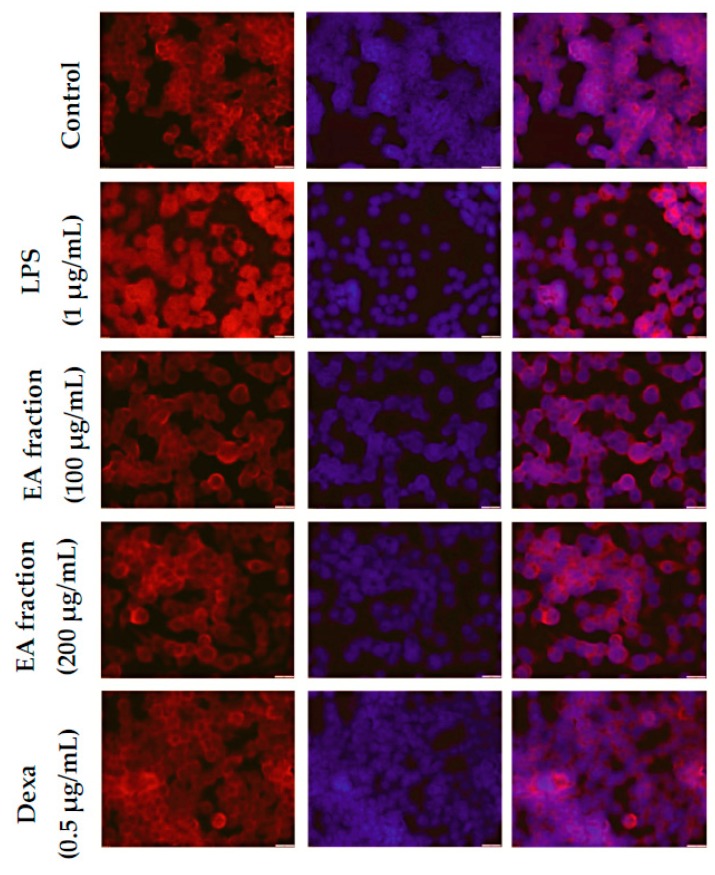
Effect of ethyl acetate fraction of *M. oleifera* extract on NF-κB signaling pathway in LPS-stimulated RAW264.7 macrophages. Cells were pretreated with ethyl acetate fraction or dexamethasone for 2 h and then stimulated with LPS to induce the inflammatory signaling cascade. Cells were fixed and processed for immunostaining with specific antibodies. Nuclei were counterstained with Hoechst stain (blue color) and observed with a fluorescent microscope (magnification of images, ×600).

The effect of the ethyl acetate fraction on NF-κB nuclear translocation in LPS-stimulated macrophages was observed using immunofluorescence staining (Figure 5). The images show that translocation of NF-κB p65 was significantly induced by LPS exposure. Treatment with ethyl acetate fraction strongly attenuated NF-κB expression and its dependent inflammatory response, similar to the results obtained from immunoblot analyses. These results concluded that the anti-inflammatory action of the ethyl acetate fraction was mediated via the NF-κB-dependent signaling pathway.

## 3. Discussion

Lipopolysaccharide is a major cell wall component of the outer membrane of Gram-negative bacteria, and stimulates immune response in mammalian cells, particularly macrophages. It plays a critical role in the pathogenesis of septic shock in animal models and induces systemic inflammation in RAW264.7 cells [20]. It binds to Toll-like receptor 4 (TLR4) and activates the cascade of proinflammatory mediators, transcription factors, and enzymes involved in NF-κB, mitogen-activated protein kinase (MAPK), and c-Jun N-terminal kinase (JNK) signaling pathways [21]. Hence, inhibitors of this signaling cascade have been considered as effective anti-inflammatory therapeutic agents.

Bioactive constituents from natural products play a major role in controlling acute and chronic diseases/disorders. They interact with various molecular components of the immune system to enhance immunity and suppress diseases which are associated with immune function [22,23]. In the present study, we screened four different solvent fractions of *M. oleifera* to study its cytotoxicity and effect on NO and proinflammatory cytokine production in LPS-stimulated macrophages. The fractions were nontoxic to macrophages at concentration <200 µg/mL, and did not affect growth or morphological features of RAW264.7 cells.

Macrophages stimulated by LPS overexpress various primary inflammatory mediators such as COX-2 and iNOS, which are involved in the synthesis of NO and PGE2. Excessive production of NO and PGE2 can trigger numerous acute and chronic inflammation-associated diseases like hypertension, cardiovascular disease, necrosis, diabetes, and joint pains [24,25]. These two molecules are key mediators of endotoxin or microbial-induced inflammation; therefore, suppression of their activity can alleviate acute and chronic inflammation. In the present investigation, we observed a concentration-dependent decrease in the level of NO and PGE2 in LPS-stimulated macrophages when treated with *M. oleifera* ethyl acetate fraction and the positive control drug, dexamethasone. The results suggested that the *M. oleifera* ethyl acetate fraction played a key role in suppressing NO and PGE2 production during inflammation. 

Proinflammatory cytokines, including TNF-α and IL-1β, secreted as a consequence of LPS activation, stimulate the production of secondary proinflammatory cytokines including IL-6 and IL-8 [24]. Among cytokines, TNF-α is vital in the initiation of the inflammatory cascade and development of chronic inflammation-associated complications [25]. The multifunctional IL-1β and IL-6 are highly expressed and are crucial in the pathogenesis of many inflammatory diseases/disorders. Their expression is predominantly regulated by the key transcription factor, NF-κB [26]. In this investigation, we found that production of proinflammatory cytokines was inhibited by treatment with *M. oleifera* fractions in a concentration-dependent manner in LPS-stimulated RAW264.7 cells.

Recently, many researchers have shown that the LPS-stimulated inflammation model is highly correlated with the activation of diverse intracellular signaling pathways, including the NF-κB and MAPK signaling cascades [27,28]. Nuclear factor-κB is one of the key transcription factors in LPS-stimulated inflammation, which regulates a number of inflammatory mediators such as iNOS, COX-2, TNF-α, IL-6, IL-8, and IL-1β [29], and plays crucial roles in the early stages of inflammatory response. The activation of NF-κB is a multistep process induced via numerous signal transduction pathways. Activated NF-κB results in an excessive production of inflammatory mediators, particularly iNOS and COX-2 [30]. iNOS and COX-2—both inducible enzymes—are responsible producing NO and PGE2, respectively, and these mediators are closely related with inflammatory cascades [31]. Treatment of LPS-stimulated macrophages with the ethyl acetate fraction of *Moringa oleifera* effectively suppressed the expression of iNOS and COX-2, which led to a decrease in the production of NO and PGE2, which are known to initiate the early stage of inflammatory pathways [32].

The IκB kinase (IKK) complex is activated by LPS in macrophages through the TLR, and phosphorylates IκBα in the cytoplasm. It consequently undergoes proteasomal degradation, resulting in the release of NF-κB p65 from the IKK complex and its translocation into the nucleus, where it stimulates the expression of target genes involved in inflammatory response [33].

To investigate the molecular mechanism of the anti-inflammatory action of the *Moringa oleifera* ethyl acetate fraction, we observed its effects on IκBα and nuclear translocation of NF-κB in LPS-stimulated RAW264.7 cells. The expression of iNOS, COX-2, and NF-κB was downregulated, while that of IκBα was upregulated upon treatment of cells with the ethyl acetate fraction. Therapeutic agents that interfere with the activation/action of NF-κB may be effective for treating various inflammation-associated diseases [34,35]. The present findings demonstrated that the anti-inflammatory potential of the ethyl acetate fraction involved suppression of NF-κB activation by preventing IκBα degradation and translocation of the NF-κB p65 protein into the nucleus.

## 4. Materials and Methods

### 4.1. Preparation of M. oleifera Extract and Isolation of Fractions

Mature *M. oleifera* leaves were harvested from Universiti Putra Malaysia Garden-No. 2. The leaf materials were verified and deposited at the Herbarium Unit, Institute of Bioscience, Universiti Putra Malaysia, with the voucher specimen number SK 1561/08. Harvested leaves were washed with water, air-dried at room temperature (24 °C) for two days, and then oven-dried at 45  °C for another two days. The dried leaves were blended with an analytical blender and stored in an airtight container for further experiments. The resulting powder was macerated with 90% ethanol in an airtight bottle for 72 h at room temperature to prepare the hydroethanolic extract. The filtrate was filtered through Whatman filter papers and condensed using a rotary evaporator. The remaining residues were freeze-dried and the extract obtained was stored at −20 °C for further cell culture and analytical experiments.

The freeze-dried extract was resuspended in 90% methanol and partitioned into various solvents—namely, hexane, ethyl acetate, butanol, and chloroform—to separate the active compounds present in the plant extract. The harvested fractions were evaporated and condensed using a rotary evaporator, and the remaining residues were freeze-dried.

### 4.2. Cell Culture and Fractions Treatment

Mouse macrophage (RAW264.7) cells were purchased from American Type Culture Collection (ATCC, Manassass, VA, USA) and were cultured in high glucose Dulbecco’s modified Eagle medium (DMEM) containing 10% fetal bovine serum and other supplements. RAW cells were subcultured and transferred into a new culture flask when they reached 80% confluence. Cultured cells were plated in 96-well microplates or 6-well culture plates with appropriate cell density and incubated overnight to allow cells to attach to the plates. *M. oleifera* extract fractions were dissolved in 0.1% dimethyl sulfoxide (DMSO) and then diluted with DMEM to obtain the desired concentration.

### 4.3. Cell Viability Assay

Cytotoxicity of the *M. oleifera* extract fractions was determined by mitochondrial-dependent reduction of MTT to purple formazan. In this experiment, RAW264.7 cells were plated at a density of 1 × 10^5^ cells in a 96-well flat-bottom plate with different concentrations of the fractions (1000, 500, 250, 125, 62.5, 31.25, and 15.625 µg/mL) in the culture media. After 24 h of incubation, MTT solution (5 mg/mL) was diluted in phosphate-buffered saline (PBS) and added to each well for further incubation for 4 h. The cell culture supernatant with MTT solution was removed and 100 µL of DMSO was added to each well to dissolve the formazan crystals. Cell viability was determined by absorbance measurement at 570 nm. The percentage of cell viability was calculated according to a previously described method [36].

### 4.4. Nitric Oxide Assay

Nitric oxide level was determined by a method based on the Griess reaction assay as we described earlier [22]. Briefly, macrophages were seeded overnight in a 6-well plate and incubated with specific concentration of *M. oleifera* extract fractions or dexamethasone for 2 h, followed by LPS (1 μg/mL) treatment. The control group was treated only with fresh medium for another 24 h. The cell culture supernatant (100 μL) was mixed with Griess reagent (0.2% naphthylethylenediamine dihydrochloride and 2% sulphanilamide in 5% phosphoric acid) and incubated for 10 min at room temperature in the dark. Finally, the absorbance was measured at 550 nm using a microplate reader. Nitrite concentrations were calculated using a standard sodium nitrite curve.

### 4.5. Measurement of Proinflammatory Cytokine Production

The level of proinflammatory cytokines (TNF-α, IL-6, and IL-1β) in the cell culture supernatant was measured using an ELISA kit (R&D Systems, Minneapolis, MN, USA) according to the manufacturer′s instructions. RAW264.7 cells were treated with different concentrations of *M. oleifera* extract fractions or dexamethasone. After 24 h, culture supernatants were harvested and stored at −20 °C for further analysis. The supernatants (100 μL) were transferred to 96-well plates coated with specific capture antibodies.

### 4.6. Western Blot Analysis

Cells were harvested after treatment with various concentrations of *M. oleifera* ethyl acetate fraction or dexamethasone. Ice-cold radioimmunoprecipitation assay (RIPA) mammalian protein extraction and lysis buffer containing protease and phosphatase inhibitor cocktails (Roche, Basel, Switzerland) was added to harvested cells to extract the protein. Cells were centrifuged at 12,000 rpm for 20 min at 4 °C. Protein concentration in the sample was estimated using a bicinchoninic acid protein assay kit, with bovine serum albumin (BSA) as the standard. Equal concentrations of protein samples were then separated by sodium dodecyl sulfate polyacrylamide gel electrophoresis using 8% polyacrylamide gel and transferred to polyvinylidene fluoride membranes. The membranes were blocked with 5% BSA and washed with PBS-Tween-20 solution (PBS-T), and then incubated with specific primary antibodies (iNOS, COX-2, NF-κB from Abcam, Boston, MA, USA and IκBα from Santa Cruz Biotechnology (Santa Cruz, CA, USA) overnight at 4 °C on a shaker. Membranes were then washed with PBS-T and incubated with horseradish peroxidase-conjugated secondary antibodies for 1 h at room temperature. After the incubation period, the membranes were washed with PBS-T and incubated with chemiluminescence substrate (Thermo Scientific, Rockford, IL, USA) to expose the blots before detection with Chemidoc™ XRS (Bio-Rad, Hercules, CA, USA). Intensity of the blots was analyzed using Bio-Rad Image Lab software (Bio-Rad, Hercules, CA, USA). 

### 4.7. Fluorescence Microscopy to Visualize NF-κB p65 Localization

RAW264.7 cells were cultured overnight on coverslips in 6-well plates and treated with various concentrations of *M. oleifera* ethyl acetate fraction or dexamethasone. After the incubation period, cells were washed (with PBS) and fixed immediately with fixation solution for 20 min at −20 °C. Fixed cells were then blocked with 1% BSA solution for 20 min at room temperature. Finally, cells were incubated with a specific dilution of primary antibody against NF-κB p65 at room temperature for 1 h, and then washed with PBS. Cells were further incubated for 1 h with secondary antibodies conjugated with fluorophores at room temperature. The cells were washed with PBS and stained with Hoechst stain (Thermo Scientific) for nuclei staining. Translocation of NF-κB p65 into the nucleus was visualized by fluorescence microscopy (Olympus, Tokyo, Japan).

### 4.8. Statistical Analysis

The statistical significance of results from different experimental groups was determined by one-way analysis of variance followed by post-hoc Tukey’s test. All experiments were performed in triplicate and the results are presented as mean ± standard deviation (SD). Differences with *p*-values of 0.05 or less were considered to be statistically significant.

## 5. Conclusions

**Figure 6:**
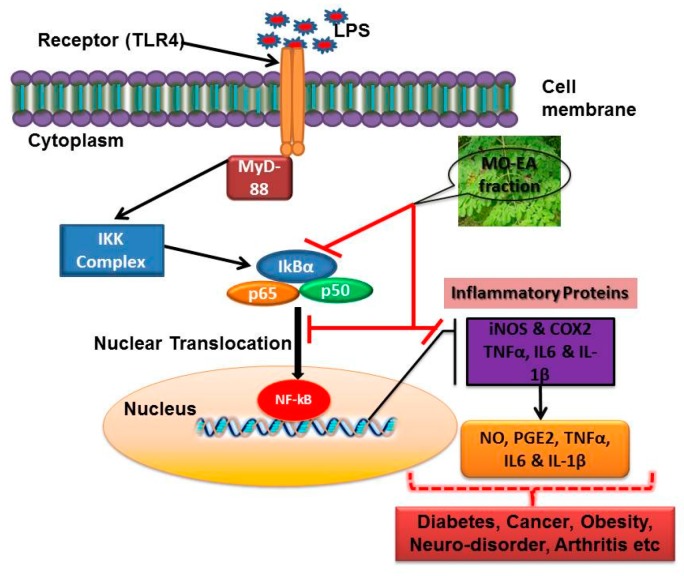
Schematic illustration of the possible inhibitory mechanism of ethyl acetate fraction of *M. oleifera* in suppressing LPS-activated inflammatory pathway.

In the present study, we demonstrated that the ethyl acetate fraction of *M. oleifera* extract has significant anti-inflammatory therapeutic value in a LPS-induced in vitro inflammatory model as shown in Figure 6. Its anti-inflammatory properties were mediated by suppression of NF-κB activation and translocation into the nucleus, as well as inhibition of the production of various inflammatory proteins in the resulting signaling cascade. Our findings help elucidate the cellular and molecular basis of the anti-inflammatory activity of *M. oleifera* ethyl acetate fraction and suggest the therapeutic potential of *M. oleifera* as a nutritional functional food for the treatment of inflammation-associated diseases and disorders (Figure 6).
